# Mesmerism

**Published:** 1890-08

**Authors:** 


### HALL'S JOURNAL OF HEALTH

TRUTH DEMANDS NO SACRIFICE; ERROR CAN MAKE NONE.

Vol. 37. AUGUST, 1890. No. 8.

### MESMERISM.

More than two hundred years ago, Frederick Anthony Mesmer, a native of Mersburg, Germany, then an undergraduate of medicine, at Vienna, made the discovery of a subtile force inherent in man, invisible, intangible, yet powerful and under favorable conditions capable of being exerted in such manner as to render certain sensitive persons, during its exercise, subservient to the will of the demonstrator, at least so far as to seem to be correspondingly affected in all the ordinary senses, and reaching beyond them into the spiritual, to form a part, so to speak, of his mentality or soul force.

The instrument which led to this discovery, was the ordinary steel magnet, whose powers of attraction and cohesion, though past dispute, have never been traced to an indisputable cause. It was the theory of Mesmer that the universe is pervaded by an element more subtile than the air, which penetrates all bodies and to which the nervous systems of all animals respond as naturally as the eye to light. In his early experiments, Mesmer made use of the magnet as a means to their success, but eventually dispensed with it as quite unnecessary. Having firmly established his conclusions by repeated demonstrations and applied his discovery to the cure of certain nervous diseases, with astonishing effect, Mesmer, in 1777, memorialized the Academy of Sciences at Paris, the Royal Society of London and the Academy of Berlin, upon his discovery, but these self-sufficient bodies of conservative unteachables, treated his claims as the vagaries of a visionary. In the following year Mesmer removed from Vienna to Paris, where he set up an establishment and achieved great renown by his marvelous cures by means of animal magnetism. His unparalleled success in the employment of purely natural means in the treatment of obstinate diseases which had long resisted the highest medical art, invested him with a wide spread popularity, which led to a scientific examination of his claims by a commission of *savants*, appointed by the French government, consisting of four well known physicians, and five members of the French Academy. This committee reported adversely to Mesmer, upon both scientific and moral grounds, and he soon afterwards found it expedient to quit France for England, where he resided for a time under an assumed name. At length, discouraged and heartbroken, he returned to Vienna, where he died in comparative obscurity, forty years after the publication of his great discovery. But this remarkable man accomplished far too much for the truth of science to die out of mind, though scientists and organized scientific bodies would fain have it so.

Other minds humble enough to confess that there were secrets of nature which they had not fathomed, or at the best, of which they had but an imperfect knowledge, restored the cause and discovery of Mesmer, and demonstrated their truth before both private and public assemblies, till they were fairly forced into recognition. Even the French Academy has reluctantly lowered its colors and acknowledged the tardy, but effectual conquest.

Does any scientist of to-day worthy the name, dispute the fact of mesmerism? As well might he dispute the fact of sunlight, or the changes of the moon. But these worthy "pull-backs" have partially succeeded in hiding their ignorance behind a new name. They would have it no longer mesmerism, but hypnotism. Thus would they despoil the great discoverer of his well earned laurels and obscure his honored memory with the mantle of their persistent folly and ignorance. But we insist that to Mesmer, and to Mesmer alone belongs the honor of the discovery of animal magnetism, its forces and possibilities, and we solemnly protest against the attempt to withhold from his name a customary honor, which, living or dead, should be accorded to him.

It is *mesmerism*, and not hypnotism, that lightens the pathway of knowledge to-day, tardily enough as through the obscurity of years. The latter term was invented by Mr. Braid, of Manchester, England, and first used by him in a treatise on Hypnotic Therapeutics, in 1853, seventy-six years after Mesmer published his theory of animal magnetism, and reduced it to practice as a powerful agent in therapeutics. Webster defines hypnotism as "A kind of sleep or somnambulism, said to be produced by animal magnetism," and since Mesmer was the first to discover animal magnetism, the honor is his and his alone. It is a niggardly soul that would pluck honor from a grave or consent to the "Martyrdom of Fame," after the power of defense had passed away with a life of fruitless endeavor and cruel disappointment.

Such indeed was Mesmer's.

